# Probing the Impact of Vacancy Diffusion on Void Dynamics at the Lithium Metal–Solid Electrolyte Interface

**DOI:** 10.1002/advs.202515827

**Published:** 2025-11-23

**Authors:** Sourim Banerjee, Bairav S. Vishnugopi, Partha P. Mukherjee

**Affiliations:** ^1^ School of Mechanical Engineering Purdue University West Lafayette IN 47907 USA

**Keywords:** lithium metal anode, solid‐state batteries, surface diffusion, vacancy diffusion kinetics, void formation

## Abstract

Lithium (Li) metal‐based solid‐state batteries (SSBs) are considered promising candidates for next‐generation energy storage due to their superior energy density and enhanced safety compared to conventional Li‐ion systems. However, their practical application is limited by challenges such as void formation at the Li‐solid electrolyte (SE) interface, which disrupts ion transport and accelerates interfacial degradation. This work investigates how the coupled effects of electro‐dissolution kinetics and surface diffusion at the Li metal surface govern the evolution of interfacial morphology during stripping. This work examines the influence of three distinct surface diffusion modes, which are terrace diffusion, step diffusion, and interlayer diffusion, on maintaining interfacial stability. In addition, how the dominant surface diffusion mechanism can overcome the contact loss due to high reaction kinetics is explored. Furthermore, the roughness of the Li metal anode surface is quantified, and the influence of different diffusion mechanisms on the evolution of the dynamic solid–solid interface is examined. The critical role of temperature in enhancing Li surface diffusivity and expanding the regime of stable contact is highlighted. By identifying distinct regimes of interface stability, this study analyzes how non‐uniform electrochemical dynamics dictate void morphology evolution and interfacial contact. These insights offer guiding principles for engineering robust Li–SE interfaces in SSBs.

## Graphical abstract

Understanding void formation at the lithium(Li) metal–solid electrolyte (SE) interface is crucial to improving interfacial stability in solid‐state batteries(SSBs). In this work, the competing electrochemical interactions, including surface diffusion modes are studied in dictating interface evolution during electro‐dissolution. A distinct surface diffusion mode for stable contact is identified, and the role of temperature and overpotential is explored.

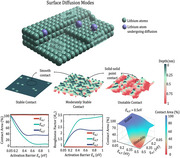

## 1 Introduction

Rechargeable lithium‐ion batteries have witnessed a market growth in deployment over the past few decades, propelled mainly by their prevalent role in powering portable electronics and electric vehicles.^[^ ^1^, ^2^, ^3^ ^]^ However, amid growing energy consumption and the urgent drive to minimize reliance on fossil fuels, solid‐state batteries (SSBs) featuring lithium (Li) metal anode have gained attention as a compelling solution for next‐generation energy storage.^[^ ^4^, ^5^, ^6^, ^7^ ^]^ Li metal electrodes are widely recognized as a desirable option in energy storage systems owing to their exceptionally low electrochemical potential (‐3.04 V vs standard hydrogen electrode), low density (0.534 g cm^−3^), and high theoretical capacity (3860 mA g^−1^).^[^ ^8^, ^9^, ^10^, ^11^, ^12^ ^]^ Furthermore, inorganic solid electrolytes (SEs) are theoretically capable of promoting significant interfacial stability, uniform deposition, and safety because of their intrinsic mechanical rigidity and non‐flammable nature.^[^ ^13^, ^14^, ^15^, ^16^, ^17^ ^]^ Although these advancements are promising, the practical deployment of SSBs remains hindered by fundamental challenges arising from the complex electro‐chemo‐mechanical interactions at the Li‐SE interfaces.^[^ ^18^, ^19^, ^20^, ^21^, ^22^, ^23^, ^24^ ^]^

The inherent solid–solid nature of the interface between the Li metal anode and the SE gives rise to mechanical and electrochemical incompatibilities, which are central to the challenges encountered in SSB performance during Li metal plating and stripping. A critical obstacle manifests as interfacial instability, driving the Li dendrite intrusion into the SE microstructure and eventually causing a short circuit.^[^ ^25^, ^26^, ^27^, ^28^, ^29^, ^30^ ^]^ Nucleation and growth of interfacial voids during Li stripping exacerbate the situation by concentrating the current distribution at limited contact regions.^[^ ^31^, ^32^, ^33^, ^34^, ^35^, ^36^ ^]^ These instabilities extensively emerge from the spatially heterogeneous electrochemical reactions occurring at the solid–solid interface.^[^ ^34^, ^35^ ^]^ A range of factors, including interfacial morphology,^[^ ^36^ ^]^ the microstructural characteristics of the SE, such as grain boundaries,^[^ ^37^, ^38^ ^]^ surface defects,^[^ ^39^ ^]^ and porous network, collectively contribute to this reaction heterogeneity. These inherent non‐uniformities also generate uneven stress distributions within both the SE and the Li metal, which can initiate thermal hotspots and provoke mechanical degradation.^[^ ^25^, ^40^, ^41^, ^42^ ^]^

Vacancy formation within the Li metal anode, initiated by interfacial metal atom dissolution during stripping, leads to a reduction in the number of electrochemically active sites. The development of interfacial voids is governed by the interplay between the rate of vacancy generation and subsequent transport into the bulk via Li diffusion. A kinetic framework based on vacancy concentration gradients is often used to evaluate the effective vacancy flux and delineate the electro‐dissolution‐limited regimes.^[^ ^43^ ^]^ Operando impedance spectroscopy has been instrumental in linking electrochemical responses to void evolution dynamics. However, direct observation of morphological changes at buried solid–solid interfaces remains experimentally challenging. In this context, X‐ray tomography has emerged as a powerful diagnostic technique for capturing real‐time interfacial degradation in SSBs.^[^ ^33^ ^]^ Studies have shown that microstructural heterogeneity, thermal gradients, and mechanical stresses strongly modulate void formation and the resulting interfacial resistance.^[^ ^44^ ^]^ To gain deeper insight into void growth mechanisms, theoretical models have employed bulk‐diffusion‐limited transport to explain Li depletion at the interface. For instance, void dynamics have been modeled analogously to bubble growth processes, enabling the prediction of critical current densities for void nucleation.^[^ ^45^ ^]^ Phase‐field approaches have been used to simulate the spatiotemporal evolution of void morphology during stripping.^[^ ^46^ ^]^ Large‐scale molecular dynamics simulations have investigated the influence of interfacial coherency on Li‐metal surface morphology during cycling.^[^ ^47^ ^]^ Furthermore, first‐principles calculations have revealed how variations in lithiophilicity at the interface affect vacancy distribution and interfacial stability.^[^ ^48^ ^]^

Interfacial contact degradation and vacancy buildup during Li stripping are key contributors to rising interfacial impedance and elevated cell overpotential. While theoretical frameworks and electrochemical signatures offer a generic overview, the intricate dynamics of stripping are governed by localized morphological irregularities and interfacial non‐uniformities. The extent of active contact area strongly influences the kinetics of the electro‐dissolution by changing the electrochemical overpotential, the spatial distribution of reaction sites, and the evolving surface profile of the Li metal. Isolated features, such as nanoscale protrusions, can act as preferential sites for filament nucleation during subsequent plating, leading to even and unpredictable growth pathways. Furthermore, elevated operating temperature enhances the surface diffusion rates, contributing to the solid–solid contact healing. That's why, a comprehensive understanding of the void evolution mechanisms is essential for mitigating rate‐dependent limitations and enhancing the mechanical and electrochemical stability of the Li–SE interface.

Surface diffusion is a fundamental phenomenon governing various physical and chemical processes on active surfaces,^[^ ^49^, ^50^, ^51^ ^]^ including phase transformation,^[^ ^52^ ^]^ crystal growth on catalysis,^[^ ^53^ ^]^ and self‐assembly.^[^ ^54^ ^]^ The self‐diffusion of single atoms plays a crucial role in the dynamic evolution of crystal surfaces^[^ ^55^, ^56^ ^]^ and has been extensively studied using experimental techniques such as field‐ion microscopy (FIM) and scanning tunneling microscopy (STM).^[^ ^57^, ^58^, ^59^ ^]^ These studies examined atomic‐scale mechanisms like hopping and exchange, along with factors influencing diffusion behaviors and their impact on epitaxial growth.^[^ ^60^, ^61^ ^]^ Theoretical models such as the terrace‐step‐kink (TSK) framework,^[^ ^62^ ^]^ step‐flow dynamics,^[^ ^63^ ^]^ and interlayer diffusion barriers^[^ ^64^ ^]^ have been developed to predict surface evolution. Notably, nonlinear approaches like the Langevin equation have provided a statistical description of kinetic roughness in growing films.^[^ ^65^ ^]^ Also, surface diffusion governs the mobility of adatoms during electrodeposition, thereby significantly shaping the emerging metal morphology.^[^ ^66^, ^67^ ^]^ Enhanced self‐diffusion along the surface facilitates smoother metal deposit growth and effectively suppresses dendritic or fractal structures, improving the cycling stability of metal‐based batteries.^[^ ^68^ ^]^

Analogous to epitaxial growth and metal electro‐deposition, surface diffusion is expected to significantly influence the void morphology and stripping dynamics of the metal electrode during electro‐dissolution reaction at the metal–SE interface. In this work, we employ a mesoscale kinetic Monte Carlo (KMC) framework to probe the coupled roles of vacancy transport, surface diffusion, ionic transport, and electrochemical reaction kinetics. Our results demonstrate how these mechanisms, particularly surface diffusion mechanisms, collectively influence surface morphology, void accumulation with the Li metal, and the spatial contact distribution at the interface. We investigate the role of the operating temperature affecting the interfacial contact loss, emphasizing its contribution to enhancing vacancy mobility and reducing void development. Additionally, we examine the role of electrochemical overpotential at the solid–solid interface on the contact loss by increasing the reaction rate at the contact points, producing more and more vacancies. Reaction hotspots, together with surface roughening, contribute to faster interfacial degradation by intensifying local current concentrations and driving accelerated void growth. Collectively, these insights establish a mechanistic basis for interpreting morphological evolution and enhancing interfacial stability in Li metal anodes for SSBs.

## 2 Methodology

During Li stripping, the interplay between electrochemical dissolution reaction, vacancy migration, surface diffusion, and the ionic transport within the SE governs the evolution of interfacial morphology. The non‐conformal contact at the Li–SE interface leads to a non‐uniform distribution of active reaction sites. When a Li atom is stripped from the interface, it leaves behind a vacant site (*V*_Li_) and an electron (*e* ^−^), while the resulting Li^+^ ion enters an interstitial site in the SE. According to Kröger–Vink notation,^[^ ^69^ ^]^ this reaction can be denoted as,

(1) $${\mathrm{Li}}_{\mathrm{Li}}^{\times }\to {\mathrm{Li}}_{\mathrm{SE}}^{+}+e^{-}+V_{\mathrm{Li}}^{\prime }$$

Vacancy nucleation occurs at the solid–solid interface during the stripping process, as Li atoms are continually removed from the active sites. While existing vacancies migrate away from the interface, new ones simultaneously form at other electrochemically active regions. The concentration and spatial distribution of these vacancies are determined by factors such as vacancy transport pathways, dominant surface diffusion modes, and the localized pattern of the metal atom distribution. The evolving nature of the Li‐SE contact, combined with the inherent stochastic nature of these processes, governs the initiation and the growth of interfacial voids.

**Figure 1:**
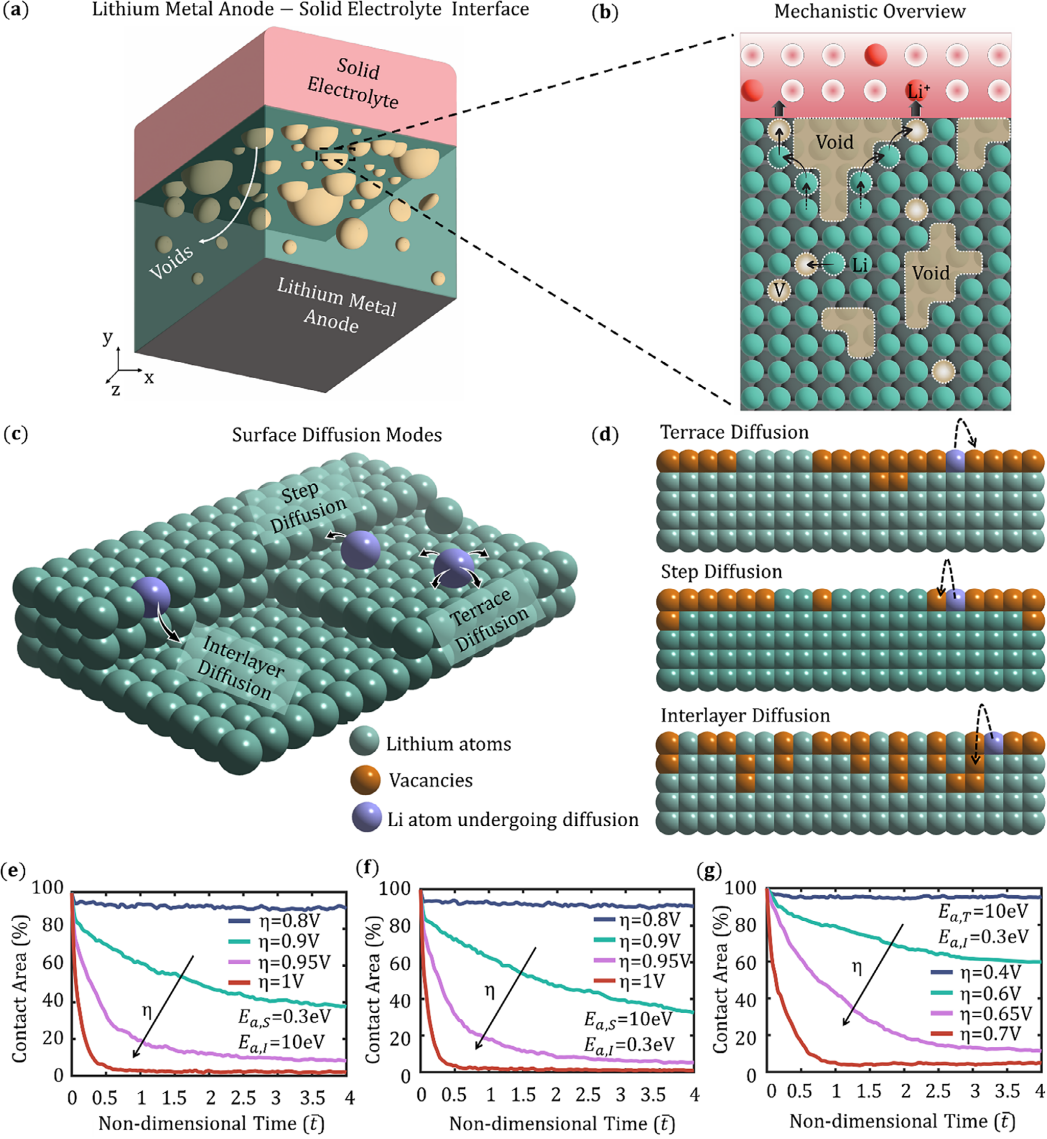
a) Schematic of a Li–SE interface containing voids inside the Li metal anode. b) Mechanistic overview in the vicinity of the Li–SE interface showing surface diffusion, bulk diffusion, reaction kinetics, and ionic transport. c) A schematic of the Li anode top layer showing all the surface diffusion modes. d) The detailed Li atom movements during three distinct modes of diffusion: terrace diffusion, step diffusion, and interlayer diffusion. Contact area evolution for increasing overpotential (η) for three different cases of surface diffusion activation potentials, e) *E*_*a*,*S*_ = 0.3 eV *E*_*a*,*I*_ = 10 eV, f) *E*_*a*,*S*_ = 10 eV, *E*_*a*,*I*_ = 0.3 eV and g) *E*_*a*,*T*_ = 10 eV, *E*_*a*,*I*_ = 0.3 eV keeping the third activation potential at base value from the literature.^[^ ^71^ ^]^

We develop a 3D KMC model to investigate the void evolution, capturing the morphological change in the Li domain and contact distribution at the interface as a function of the stripping process. In this study, Li_7_La_3_Zr_2_O_12_(LLZO) is adopted as the model SE, which is electrochemically inert with Li metal. Sulfide solid‐electrolytes, which form interphases, are not suited in this model because the ion transport properties will change the quantitative results, but would not alter the mechanism‐level trends in this work. As shown in **Figure** **1**a,b, the model incorporates four competing mechanisms occurring near the Li–SE interface as follows:

- Mechanism 1: Vacancy transport from the Li–SE interface to the bulk of the electrode.
- Mechanism 2: Surface diffusion of Li atoms at the top of the Li metal surface.
- Mechanism 3: Dissolution reaction at the electrochemically active interface is modeled based on the Butler‐Volmer reaction kinetics.^[^ ^70^ ^]^
(2) $$J=i_{0}\left(\exp\left(\frac{\alpha F}{\mathrm{RT}}\eta \right)-\exp\left(-\frac{\beta F}{\mathrm{RT}}\eta \right)\right)$$
- Mechanism 4: Li^+^ ions transport from the interface to the bulk SE.

where α and β are the charge transfer coefficients (here we consider α, β = 0.5), R is the gas constant, *T* is the operating temperature, *F* is Faraday's constant, and η is the local overpotential driving the electrochemical reactions. Also, *i*_0_ is the exchange current density for the stripping reaction, and *J* is the electrode current density.

Surface diffusion plays a critical role in shaping void morphology and the rough Li metal surface by redistributing Li adatoms along the interface, thereby smoothing local curvature variations and influencing the size, shape, and spatial evolution of voids during stripping. Figure 1c,d illustrates the three modes of surface diffusion as follows:

- Terrace diffusion: Li adatoms migrate across a flat region of the crystalline surface, known as a terrace.
- Step diffusion: Li adatoms migrate along the edge of a step on a crystal surface.
- Interlayer diffusion: Li atom migration from one atomic layer to another layer, typically crossing a step edge. Interlayer diffusion can occur in either a downward (descending) or upward (ascending) direction.

As oxidation reactions proceed, vacancies are progressively driven from the interface into the bulk of the anode. The reaction rate (*k_R_*) at each lattice site along the Li‐SE interface is defined as,

(3) $$k_{R}=\left\{\begin{array}{c} 0\\ k_{1} \end{array}\right.\begin{array}{c} \mathrm{if}\;\mathrm{vacancy}\\ \mathrm{if}\;\mathrm{Li}\;\mathrm{atom} \end{array}$$

If a lattice site on the top surface of the electrode is vacant, which means a Li atom doesn't occupy it, the reaction rate is set to *k_R_* = 0. Conversely, if a Li atom occupies the site, the reaction rate corresponds to the intrinsic reaction rate, *k_R_* = *k*_1_, denoted as, and is defined as follows:

(4) $$k_{1}=\frac{Ja^{2}}{F}N_{a}$$

here *a* is the lattice constant for Li, which is 3.5Å and *N_a_* is the Avogadro constant. Based on transition state theory,^[^ ^72^ ^]^ the bulk diffusion of Li atoms obeys the Arrhenius equation. The Li atom bulk‐diffusion rate (*k_D_*) is given by,

(5) $$k_{D}=\nu \exp\left(-\frac{E_{a}}{k_{b}T}\right)$$

In this expression, ν denotes the hopping frequency^[^ ^73^ ^]^ (ranging between 10^12^ s^−1^and10^13^ s^−1^), *k*_b_ is the Boltzmann constant, and *E_a_* is the corresponding activation energy for a Li atom transitioning between adjacent lattice sites within the Li metal, taken as 0.41eV^[^ ^71^, ^74^ ^]^ in this study. In our model, vacancy diffusion is treated as occurring in the direction opposite to that of Li atom hopping. Similarly, the surface diffusion rate (*k_SD_*) of atoms at the Li metal surface is also evaluated as follows,

(6) $$k_{SD}=\nu \;\exp\left(-\frac{E_{a}}{k_{b}T}\right)$$

For the three distinct modes of surface diffusion, the values of the activation barriers are considered from the literature. Activation energy for terrace diffusion (*E*_*a*,*T*_) is 0.3 eV, activation energy for step diffusion (*E*_*a*,*S*_) is 0.15 eV and activation energy for interlayer diffusion (*E*_*a*,*I*_) is 0.5eV.^[^ ^71^ ^]^ The Li^+^ ion diffusion rate inside the SE, denoted as *k_T_*, representing the movement of ions between adjacent lattice sites, is determined as,

(7) $$k_{T}=\nu \;\exp\left(-\frac{E_{a}}{k_{b}T}\right)$$

Here, the activation energy pertains to the ion migration within the SE, and is assumed to be 0.35 eV.^[^ ^75^, ^76^ ^]^ To incorporate temperature‐dependent effects, we further adapt the Arrhenius expressions (Equations (5), (6), (7)) to account for the temperature‐sensitive parameters, including the ion diffusion rate in the SE (*k_T_*), Li bulk diffusion rate (*k_D_*), and surface diffusion rate (*k_SD_*) as given below,

(8) $$X_{T}=X\exp\left(-\frac{E_{a,X}}{k_{b}}\left(\frac{1}{T}-\frac{1}{T_{0}}\right)\right)$$

here, *X_T_* represents the value of the temperature‐dependent parameters at an arbitrary temperature T, and X represents the value of that parameter at the reference temperature *T*_0_ (27 °C in this study). For cubic LLZO, the reported room‐temperature Li⁺ diffusivity lies in the range 10^−13^ m^2^ s^−1^ to 10^−12^ m^2^ s^−1^.^[^ ^77^ ^]^ By comparison, bulk vacancy diffusion in Li metal is typically 10^−15^ to 10^−14^ m^2^ s^−1^,^[^ ^78^ ^]^ at least an order of magnitude slower than Li⁺ motion in the solid electrolyte. In our model, the activation energy for Li⁺ diffusion in the electrolyte is 0.35 eV, while bulk vacancy diffusion in Li metal is 0.41 eV. This contrast in both diffusivity and activation energy shows that Li⁺ transport in LLZO is not the rate‐limiting step for the interfacial morphology studied. We therefore do not visualize Li⁺ concentration fields inside the electrolyte. The electrolyte behaves effectively as a single‐ion conductor with high Li⁺ mobility, so the interfacial Li⁺ activity remains close to its bulk value. As a result, charge transfer at true contact points together with mass transport on the Li side, namely vacancy and surface diffusion, governs the evolution of contact area, void fraction, and roughness. Within the operating window considered here the electrolyte does not accumulate Li⁺ to levels that would hinder charge transfer or alter the morphology‐controlling mechanisms, which motivates our focus on lithium‐metal morphology during electro‐dissolution.

In our computational framework, the simulation domain is a 50 ⨉ 50 ⨉ 50 lattice supercell, representing a cuboid volume with a side length of 17.5 nm. Periodic boundary conditions are imposed along the x and z directions perpendicular to the electrode depth. The simulation timestep is determined using the following KMC formulation,

(9) $$\Delta t=-\frac{\ln\left(r\right)}{K}$$

Here, r is a randomly generated number, and K represents the total sum of all reaction and transport rates at a given timestep. A non‐dimensional time ($\bar{t}$) is introduced to characterize the progress of Li stripping, and is computed as,

(10) $$\bar{t}=\left(t+\Delta t\right)\times K_{v_{0}}$$

where $K_{v_{0}}$ denotes the bulk diffusion rate of Li metal at room temperature, calculated based on the bulk diffusivity of Li, *D*_Li_ = 2 × 10^−14^ m^2^ s^−1^ as given by

(11) $$K_{v_{0}}=D_{\mathrm{Li}}/a^{2}$$

Details of the KMC modeling approach are provided in Figure S1 (Supporting Information) and a summary of all model parameters is presented in Table S1 (Supporting Information).

## 3 Results and Discussion

### 3.1 Dynamic Li–SE Contact Evolution during Electrochemical Stripping

The dynamic evolution of the contact area at the Li–SE interface as a function of reaction rate is shown in Figure 1e–g for three cases of surface diffusion activation barriers. In each KMC time step, the contact area is calculated from the number of lattice contacts between the metal domain and the SE domain. As time evolves, vacancies are generated at the top surface of the metal electrode due to electrochemical stripping reaction and diffuse through the depth of the electrode due to vacancy concentration gradient between the Li–SE interface and the bulk electrode. The final void morphology and the solid–solid contact distribution strongly depend on the atomic surface diffusion modes, vacancy diffusion, and electro‐dissolution reaction. The base values of the surface diffusion activation energies are *E*_*a*,*T*_ = 0.3 eV, *E*_*a*,*S*_ = 0.15 eV and *E*_*a*,*I*_ = 0.5 eV, as reported in the literature,^[^ ^71^ ^]^ and are used in each simulation unless otherwise specified. The surface diffusion activation barriers are defined as *E*_*a*,*S*_ = 0.3 eV, *E*_*a*,*I*_ = 10 eV for Figure 1e, which means the impact of interlayer diffusion is negligible. With an increase in reaction rate, i.e., overpotential η from 0.8 V to 1 V, the contact loss increases due to more vacancy injection resulting from the electrochemical reaction. Similarly, in Figure 1f,g the values of the activation barriers are *E*_*a*,*S*_ = 10 eV, *E*_*a*,*I*_ = 0.3 eV and *E*_*a*,*T*_ = 10 eV, *E*_*a*,*I*_ = 0.3 eV, which implies the impact of step diffusion and terrace diffusion are negligible respectively. From the plots, it is clear that the absence of interlayer diffusion and step diffusion does not significantly affect the contact evolution. In the absence of terrace diffusion, the contact area evolution is very drastic as the contact area reaches 5% for η = 0.7 V, which is less than η = 1 V in the other contact evolution plots. This analysis suggests that terrace diffusion has the most significant impact on maintaining a better Li–SE contact compared to the other two surface diffusion mechanisms.

**Figure 2:**
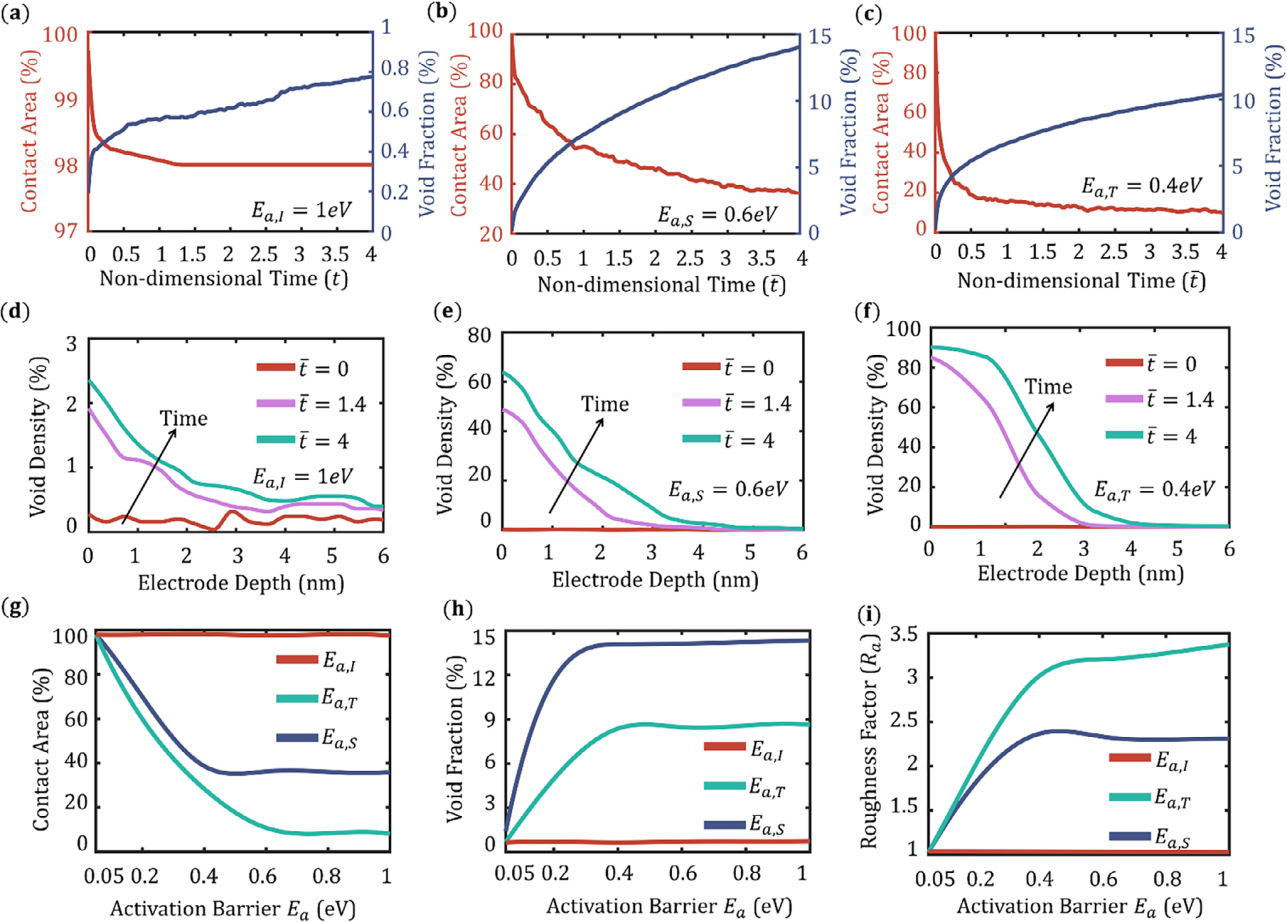
Contact area and void fraction evolution for three cases of surface diffusion modes a) *E*_*a*,*I*_ = 1 eV, b) *E*_*a*,*S*_ = 0.6 eV, c) *E*_*a*,*T*_ = 0.4 eV. d–f) Void density variation along the depth of the Li metal electrode for the above‐mentioned surface diffusion activation potential, respectively. g) Contact area, h) void fraction, i) roughness factor evolution as a function of surface diffusion activation energies.

The dynamic evolution of contact area and void fraction within the Li metal electrode is illustrated in **Figure** **2**a–c. This study investigates three distinct surface diffusion scenarios characterized by different activation energies: interlayer diffusion *E*_*a*,*I*_ = 1.0 eV (Figure 2a), step diffusion *E*_*a*,*S*_ = 0.6 eV (Figure 2b), and terrace diffusion *E*_*a*,*T*_ = 0.4 eV (Figure 2c). At each KMC time step, the void fraction is evaluated based on the number of vacancies generated within the Li metal domain. In Figure 2a, the elevated interlayer diffusion barrier suppresses vertical atomic transport, thereby promoting terrace and step diffusion. This results in minimal void formation and negligible contact loss, with the interfacial contact area remaining close to 100% and the void fraction staying below 1% throughout the stripping process.

In contrast, Figure 2b,c exhibit more pronounced contact degradation, with contact area decreasing to approximately 40% and 10%, respectively. These cases correspond to lower interlayer diffusion barriers, enabling enhanced atomic migration between layers and resulting in the accumulation of vacancies at the Li–SE interface. Notably, the extent of contact loss in Figure 2c is greater than that in Figure 2b, indicating that dominant step diffusion (in conjunction with interlayer diffusion) contributes more significantly to contact loss than terrace diffusion. Step diffusion tends to promote vacancy clustering near the interface, facilitating either electro‐dissolution of Li atoms or downward atomic diffusion via interlayer pathways. Conversely, dominant terrace diffusion in Figure 2b leads to a more dispersed vacancy distribution at the interface, which in turn supports continued interlayer diffusion and ultimately results in a higher void fraction compared to the step diffusion‐dominant case.

Figure 2d–f depicts the temporal evolution of void density for the three surface diffusion cases. Void density is calculated by counting the number of vacancies present at each lateral layer parallel to the Li–SE interface. Over time, the region of condensed void density progressively shifts deeper into the electrode, driven by a combination of vacancy injection originating from the electrochemical stripping reaction and subsequent vacancy transport. Both surface diffusion mechanisms and bulk diffusivity influence the extent and nature of this transport. In Figure 2d, where terrace diffusion is dominant, the void density remains below 3% throughout the simulation, reflecting the stabilizing effect of lateral vacancy spreading. In contrast, Figure 2e shows moderate void accumulation near the Li–SE interface due to the interplay between terrace and interlayer diffusion. Finally, Figure 2f exhibits significant void accumulation localized near the interface, a direct consequence of dominant step and interlayer diffusion pathways that promote rapid vacancy clustering. This accumulation ultimately accelerates interfacial degradation and leads to substantial contact loss.

### 3.2 Influence of Surface Diffusion Modes on Contact Area, Void Fraction, and Surface Roughness

The dynamic interplay between the various surface diffusion modes strongly governs the interfacial stability of the Li‐SE interface and the void morphology. Figure 2g–i shows the impact of surface diffusion modes, i.e., interlayer diffusion, terrace diffusion, and step diffusion, on the interfacial microstructural attributes such as contact area, void fraction, and roughness. The roughness factor *R_a_* defined as below:

(12) $$R_{a}=\frac{1}{L_{x}\times L_{z}}\sqrt{\sum_{i=1}^{L_{x}\times L_{z}}y_{i}^{2}}$$

Where, L_x_ and L_z_ are the number of cubic shells along x and z axes respectively. y_i_ denotes the depth of the Li metal surface from the Li‐SE interface. In Figure 2g, the three different contact area trends are shown as a function of activation energies *E*_*a*,*I*_, *E*_*a*,*S*_ and *E*_*a*,*T*_. We can see that *E*_*a*,*I*_ has the least impact on the contact area with the given base values *E*_*a*,*T*_ = 0.3 eV, *E*_*a*,*S*_ = 0.15 eV, which means terrace diffusion and step diffusion together can maintain a stable contact at the interface and produce the least void fraction and rough surface. Now, with an increase in *E*_*a*,*T*_, the step diffusion, along with interlayer diffusion, is dominant, resulting in rapid contact loss due to high clustering of vacancies at the interface, which leads to point contacts and increased interfacial resistance. In Figure 2h, with an increase in *E*_*a*,*S*_, terrace diffusion combined with interlayer diffusion facilitates void injection inside the Li metal domain, resulting in a higher void fraction compared to the step diffusion case. In short, with significant interlayer diffusion, high terrace diffusion leads to minimum contact loss and a high void fraction. In contrast, high step diffusion cases result in high contact loss and a lower void fraction inside the Li metal. In Figure 2i, the roughness increases with the absence of terrace surface diffusion due to clustering of vacancies at the top layers of the Li‐SE interface. On the contrary, the lack of interlayer diffusion produces a smoother surface due to less atom diffusion from the interface to the bulk. The values of the contact area, void fraction and surface roughness factors are sensitive to the activation energy barriers considered in the system due to the inherent stochastic nature of the KMC model. A detailed sensitivity analysis of the energy barriers are done and shown in Figure S4 (Supporting Information).

### 3.3 Competing Impact of Surface Diffusion Modes on Contact Area

**Figure 3:**
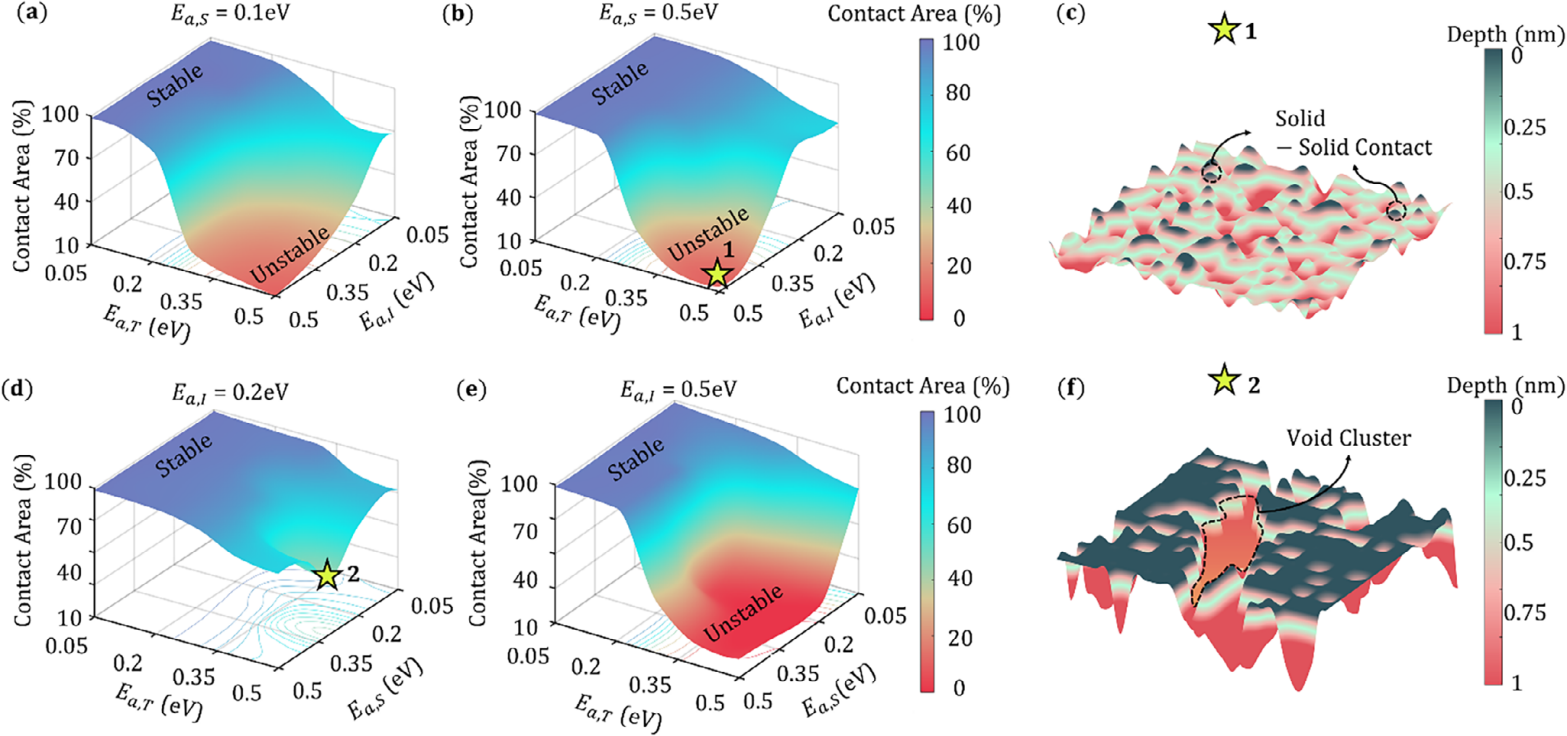
a,b) Contact area maps as a function of *E*_*a*,*T*_ and *E*_*a*,*I*_ for *E*_*a*,*S*_ =0.1 eV and *E*_*a*,*S*_ = 0.5 eV. c) Rough surface of Li metal for the surface diffusion activation barriers *E*_*a*,*T*_ = *E*_*a*,*S*_ = *E*_*a*,*I*_ = 0.5 eV. d,e) Contact area maps as a function of *E*_*a*,*T*_ and *E*_*a*,*S*_ for *E*_*a*,*I*_ = 0.2 eV and *E*_*a*,*I*_ = 0.5 eV. f) Rough surface of Li metal for the surface diffusion activation barriers *E*_*a*,*T*_ = 0.5 eV, *E*_*a*,*S*_ = 0.3 eV, *E*_*a*,*I*_ = 0.2 eV.

**Figure** **3** shows the competing effect of the surface diffusion modes on the final contact area and how that will impact the top rough surface of the Li metal domain. In Figure 3a,b, the contact area is plotted as a function of *E*_*a*,*T*_ and *E*_*a*,*I*_ for high step diffusion rate (*E*_*a*,*S*_ = 0.1 eV) and low step diffusion rate (*E*_*a*,*S*_ = 0.5 eV). High terrace diffusion (*E*_*a*,*T*_ < 0.2 eV) always stabilizes the interfacial Li‐SE contact despite of the other surface diffusion modes acting on the interface. As we move the terrace diffusion from dominant to suppressed (*E*_*a*,*T*_ = 0.5 eV) there is a clean transition from stable (70%) to unstable (10%) contact area due to suppressed interlayer diffusion (*E*_*a*,*I*_ > 0.2 eV) in both the cases of step diffusion. In Figure 3b when all the surface diffusion is suppressed (*E*_*a*,*T*_ = *E*_*a*,*S*_ = *E*_*a*,*I*_ = 0.5 eV) only bulk diffusion acts as a vacancy diffusion mechanism. The corresponding Li top surface profile is shown in Figure 3c, where the formation of point contacts can be observed. The Li metal top morphology, solid‐solid contact, void fraction and void density can vary due to the sole effect of bulk diffusion when the surface diffusion is constant (Figure S2, Supporting Information). In Figure 3d,e the competitive effect of terrace diffusion and step diffusion are shown for high interlayer diffusion rate (*E*_*a*,*I*_ = 0.2 eV) and low interlayer diffusion rate (*E*_*a*,*I*_ = 0.5 eV). There is a highest contact loss regime in the Figure 3d for the value of *E*_*a*,*S*_ = 0.3 eV combined with high interlayer diffusion (*E*_*a*,*I*_ = 0.2 eV). This can be explained by the Li rough surface profile in Figure 3f, where the vacancy clustering at the top layer leads to significant surface diffusion of Li atoms from the top surface to the subsequent atomic layer due to interlayer diffusion. But with low interlayer diffusion (*E*_*a*,*I*_ = 0.5 eV), the interfacial contact becomes very unstable as bulk diffusion mechanism comes into effect.

Our LLZO‐based model captures the qualitative trend that is faster terrace diffusion redistributes adatoms and vacancies to help maintain interfacial contact during stripping, although this effect is less dominant in sulfide SEs where interphase‐mediated transport becomes a major pathway. For example, in SEs such as Li_10_GeP_2_S_12_ (LGPS) and argyrodite Li_6_PS_5_Cl (LPSCl), ion‐conducting and electron‐blocking interphases rich in Li_2_S, Li_3_P, and halides form and evolve with current, and their higher electrolyte conductivity lowers overpotential and vacancy injection, shifting control away from terrace migration.^[^ ^79^, ^80^, ^81^, ^82^, ^83^ ^]^ Adsorbates and local fields at solid–solid interfaces can also modify Li adatom diffusion barriers relative to clean Li, and while these changes are most relevant for interphase‐forming sulfides and outside the present scope, our model targets LLZO without a chemical interphase and can be extended by adding a thin finite‐conductivity interphase layer to partition the overpotential. However, modeling such systems is beyond the scope of this study.^[^ ^84^, ^85^, ^86^ ^]^

### 3.4 Role of Overpotential on Void Growth

**Figure 4:**
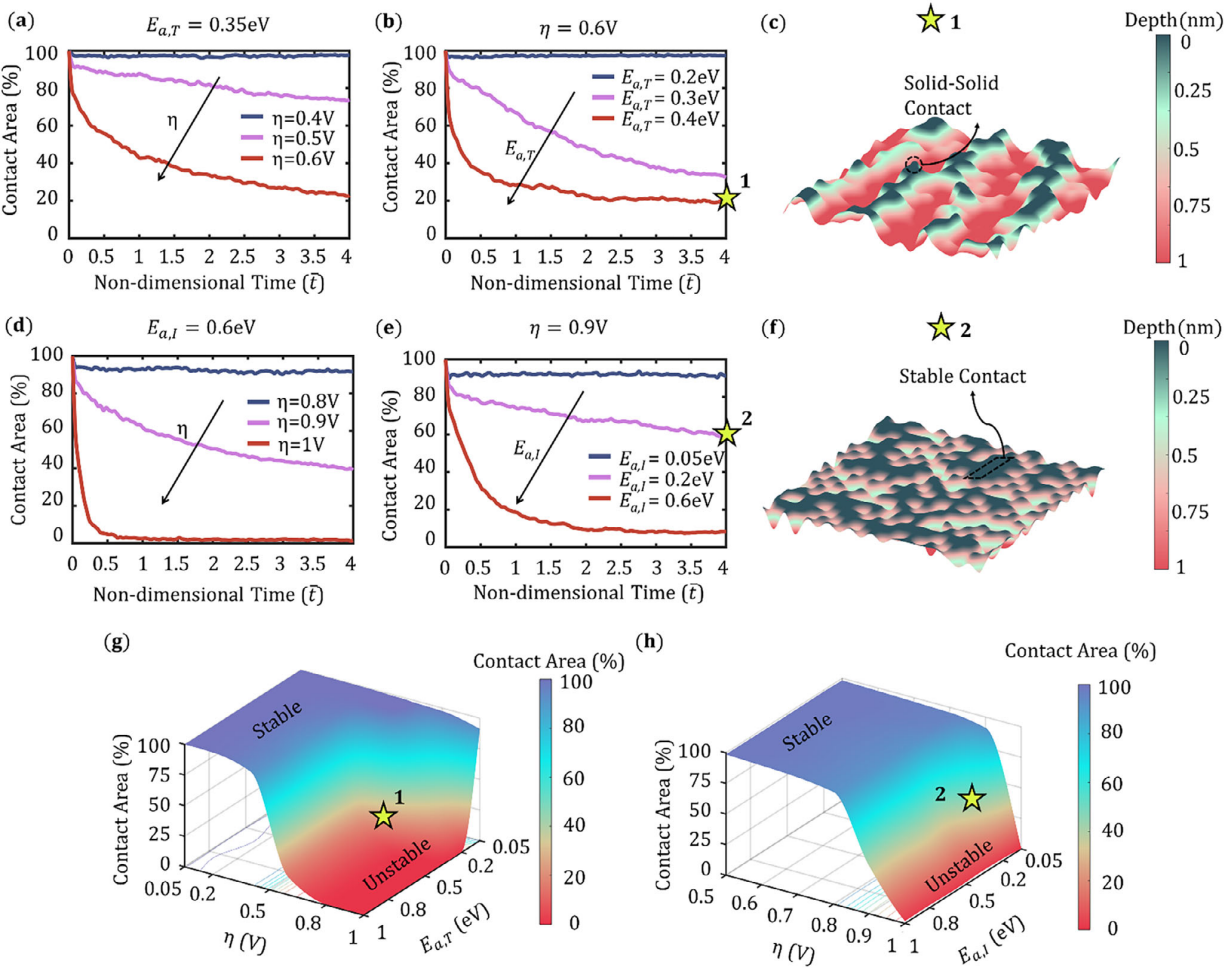
a,b) Contact area evolution as a function of *E*_*a*,*T*_ and η for *E*_*a*,*T*_ = 0.35 eV and η = 0.6 V respectively. c) Li metal rough surface for *E*_*a*,*T*_ = 0.4 eV, η = 0.6 V. d,e) Contact area evolution as a function of *E*_*a*,*I*_ and η for *E*_*a*,*I*_ = 0.6 eV and η = 0.9 V respectively. f) Li metal rough surface for *E*_*a*,*I*_ = 0.2 eV, η = 0.9 V. Contact area maps as a function of g) *E*_*a*,*T*_ and η and h) *E*_*a*,*I*_ and η.

The overpotential between the Li metal anode and the SE is governed by both the applied current and the extent of interfacial solid–solid contact. According to Equation (2), an increase in overpotential (η) enhances the rate of the electro‐dissolution reaction, which in turn accelerates vacancy generation and promotes contact loss at the Li–SE interface. As demonstrated earlier, the terrace surface diffusion mode contributes to maintaining stable solid–solid contact. **Figure** **4** illustrates the combined effects of overpotential and terrace diffusion on the evolution of interfacial contact. In Figure 4a, the contact area is plotted along stripping progress for three values of overpotential (η = 0.4 V, 0.5 V, 0.6 V) under moderate terrace diffusion conditions (*E*_*a*,*T*_ = 0.35 eV). As the overpotential increases, the rate of electrochemical stripping intensifies, resulting in more pronounced contact degradation. In Figure 4b, the terrace diffusion activation energy (*E*_*a*,*T*_) is varied from 0.2 to 0.4 eV under a constant overpotential (η = 0.6 V). A significant decline in contact area is observed with increasing *E*_*a*,*T*_, indicating that insufficient terrace diffusion impedes interfacial healing and accelerates contact loss. The corresponding Li metal top‐surface morphology for the case *E*_*a*,*T*_ = 0.4 eV and η = 0.6 V is presented in Figure 4c, revealing the formation of discrete solid–solid point contacts. Similarly, Figure 4d shows the evolution of contact area as a function of stripping for three overpotential values (η = 0.8, 0.9, 1.0 V) at a constant interlayer diffusion activation energy (*E*_*a*,*I*_ = 0.6 eV). As η increases, the contact area drops markedly, despite unchanged surface diffusion kinetics. However, further increase in the interlayer diffusion barrier reduces surface diffusivity, as shown in Figure 4e. The Li surface morphology for the case *E*_*a*,*I*_ = 0.2 eV and η = 0.9 V is depicted in Figure 4f, revealing substantial interfacial void formation and roughening. Figure 4g,h presents contact area maps as functions of η versus *E*_*a*,*T*_ and η versus *E*_*a*,*I*_, respectively. In Figure 4g, a clear transition from stable to unstable contact is observed as the terrace diffusion rate decreases from high (*E*_*a*,*T*_< 0.2 eV) to low (*E*_*a*,*T*_> 0.2 eV). Notably, even when terrace diffusion is suppressed (*E*_*a*,*T*_ > 0.8 eV), a shift from stable to unstable contact still occurs when the overpotential rises from low (η < 0.4 V) to high (η > 0.4 V). Similarly, Figure 4h shows a distinct transition from stable to unstable contact as *η* increases beyond 0.7 V, while variations in the interlayer diffusion barrier *E*_*a*,*I*_ have comparatively less influence on the contact stability.

We selected an overpotential window of 0.4 to 1.0 V to encompass the polarization commonly observed during lithium stripping at Li‐LLZO interfaces, ranging from well‐contacted to contact‐limited states. In practice, the measured voltage drop in symmetric or full cells combines charge‐transfer losses with ohmic and contact contributions, so the same current density can correspond to very different polarizations depending on interfacial chemistry, true contact area, and stack pressure.^[^ ^81^, ^82^, ^87^, ^88^ ^]^ Using a Butler‐Volmer description for the kinetic part and an area‐specific resistance term for transport and constriction, tens of millivolts are sufficient to sustain lower than 1 mA cm^−2^ currents when the exchange current density is high and the interface is well prepared, whereas several hundred millivolts up to about 1 V are frequently associated with degraded contact, limited wetting, or transient void growth during stripping. Therefore, the chosen window serves two purposes, at the lower end it overlaps with routine operation in optimized cells, and at the upper end it stress‐tests the model in the contact‐limited regime where void nucleation and coalescence are most active. This range is thus representative of realistic conditions and enables clear mapping between polarization, morphology, and stability. It also facilitates comparison to published symmetric‐cell data across interface treatments and pressures. Morphology and stability vary with the applied current density because it sets the vacancy injection rate while bulk and surface diffusion provide relaxation. At low applied current density, diffusion keeps up, delaying void nucleation, preserving contact, and limiting roughness. At moderate applied current density, voiding emerges with sensitivity to surface mobility barriers. At high applied current density, injection outpaces vacancy removal rate leading to voids coalescing, current constrictions, and polarization rises, reinforcing contact loss. Thus, instability thresholds shift with applied current density, and terrace diffusion remains the key stabilizer.

### 3.5 Surface Roughness Analysis

**Figure 5:**
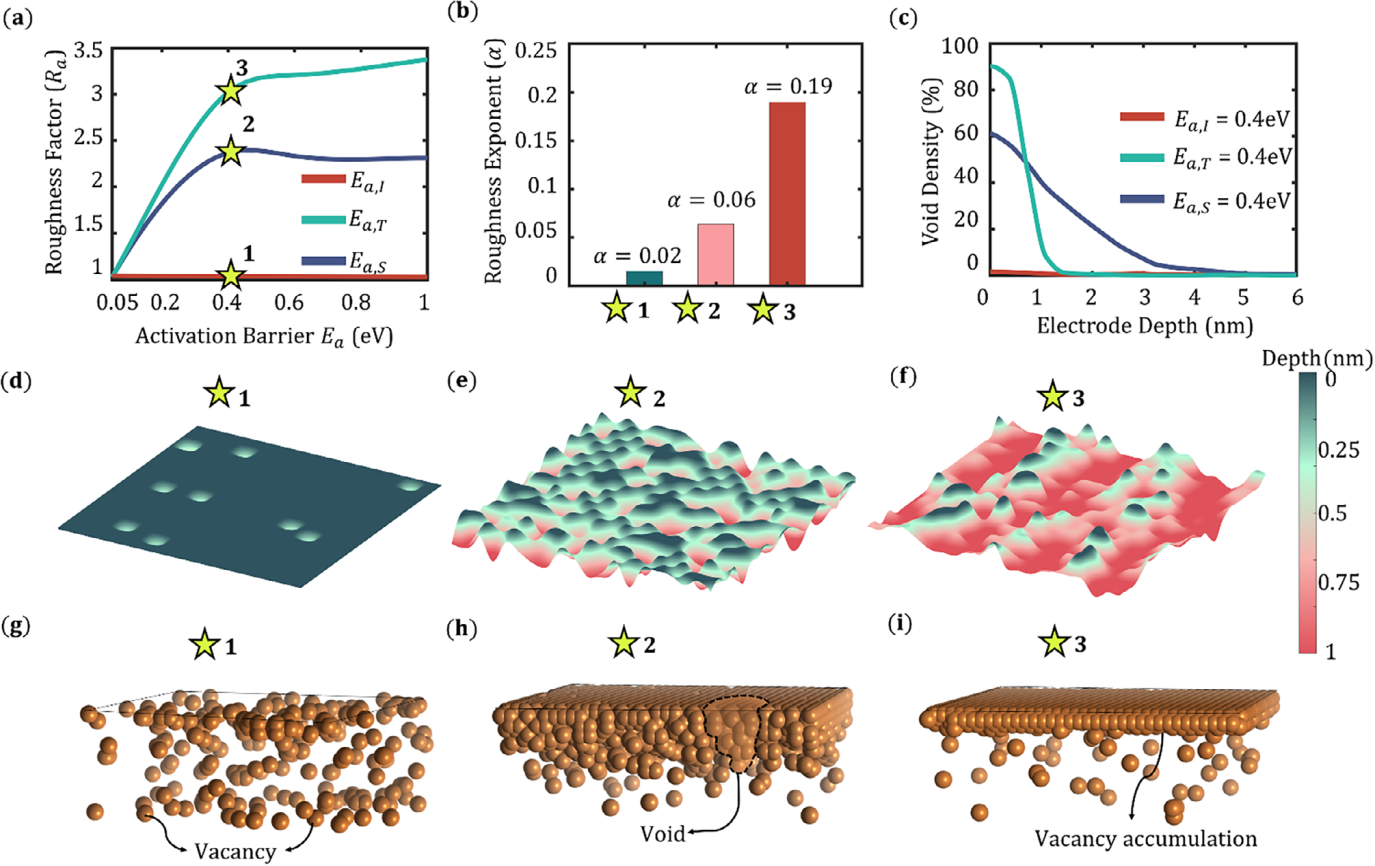
a) Roughness factor (*R_a_*) evolution as a function of activation energies for terrace, step, and interlayer diffusion. b) The value of the roughness exponent (α) for rough surfaces from smooth surface to rough surface. c) Void density variation for three cases of activation potential *E*_*a*,*I*_ = 0.4 eV, *E*_*a*,*S*_ = 0.4 eV, *E*_*a*,*T*_ =0.4 eV. d–f) Li metal rough surfaces at the end of stripping and three distinct void morphologies, i.e., g) distributed vacancies, h) small void clusters, i) big void clusters at the top layers of the Li domain corresponding to *E*_*a*,*I*_ = 0.4 eV, *E*_*a*,*S*_ = 0.4 eV, *E*_*a*,*T*_ =0.4 eV respectively.

Morphological changes at the Li metal interface during stripping are strongly influenced by interfacial roughness, and that's why it is crucial to characterize the rough surface profiles. Our findings demonstrate that variations in surface diffusion mechanism, electrochemical overpotential, and temperature lead to pronounced alterations in surface topology, contributing to void formation and loss of interfacial contact. In **Figure** **5**a, the surface roughness factors (*R_a_*) are calculated using Equation (12) for three cases of surface diffusion mode variations, and the results are shown. There is a significant increase in roughness when terrace diffusion is absent, due to vacancy clustering. And in contrast, the absence of the interlayer diffusion mode doesn't affect *R_a_* because of low atomic transport from the interface to the bulk. To further analyze the surface profiles marked in the Figure 5a we have computed the normalized “Structure Factor”^[^ ^89^ ^]^ *S*(*q*) from the top surface profiles given in Figure 5d–i. The formulation is given below,

(13) $$S\left(q\right)=\frac{1}{N}\langle \sum_{i=1}^{N}\sum_{j=1}^{N}e^{i\vec{q}•\left(\vec{r_{i}}-\vec{r_{j}}\right)}\rangle$$

here, *N* is the number of total atoms present in the domain, *q* is the reciprocal space vector or a wavevector, and *r_i_* and *r_j_* represents the spatial position of the atoms of the surface. For a self‐affine interface, the structure factor *S*(*q*) scales with wavevector *q* for an extensive range of values of *q*.^[^ ^90^ ^]^ The exact scaling relation is given as,

(14) $$S\left(q\right)∼q^{-\alpha }$$

where, α is a positive constant defined as the roughness exponent. In Figure 5b, the calculated α values are shown for three distinct rough surfaces. High roughness constant (α = 0.19) denotes highly uneven and irregular Li metal surface created when the terrace diffusion is absent in the Li domain. Whereas, the absence of step diffusion and interlayer diffusion produces a smoother surface (α = 0.06 and α = 0.02, respectively) due to significant atom diffusion towards the interface from the bulk, replacing the vacancies, providing a stable interfacial contact. In Figure 5c the void density for the three cases of activation barriers is plotted with the electrode depth. As shown in the plot, as we suppress the surface diffusion modes from interlayer to step diffusion to terrace diffusion, the void density at the interface changes from zero gradient to a higher gradient of void density. In the case of *E*_*a*,*T*_ = 0.4 eV, the vacancy accumulation at the interface is very high, leading to high contact loss. In contrast, in the case of *E*_*a*,*I*_ = 0.4 eV, the vacancy accumulation is negligible. In Figure 5d–f, the Li top surfaces and in Figure 5g–i the vacancy distributions are shown for the three cases of surface diffusion barriers. When the terrace and interlayer diffusion are dominant (marked as 1), the vacancy diffusion is much higher, leading to the least contact loss and a smooth anode surface (Figure 5d,g). In contrast, when terrace and interlayer diffusion are dominant (marked as 2), there is moderate contact loss and formation of void clusters due to the combined effect (Figure 5e,h). However, the combined step diffusion and interlayer diffusion cause the maximum contact loss, resulting in a high accumulation of vacancies at the interface, which leads to solid–solid contact points (Figure 5f,i).

### 3.6 Effect of Temperature

**Figure 6:**
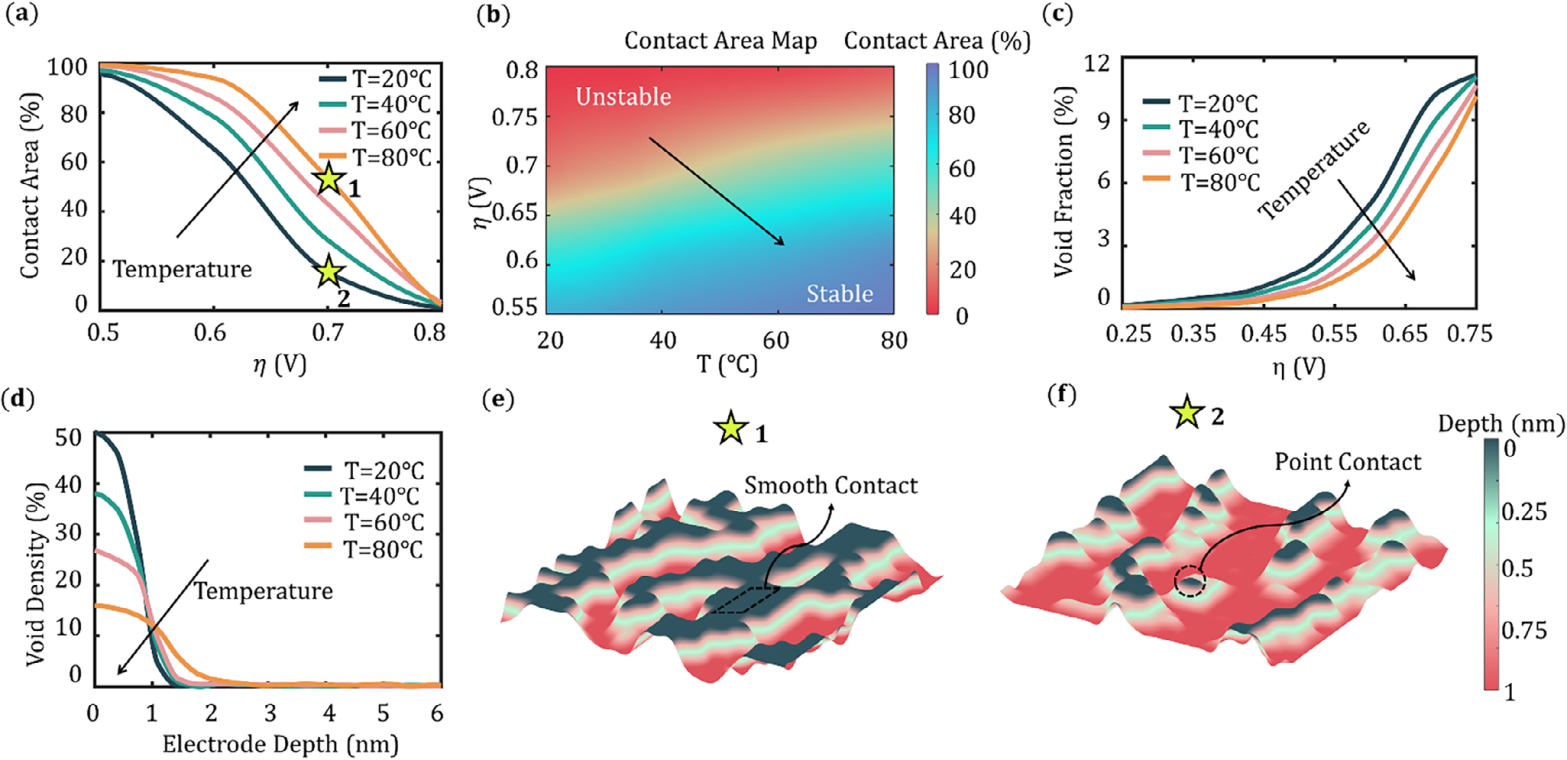
a) Contact area variations as a function of η for temperature *T* = 20 °C to *T* = 80 °C. b) Contact area map as a function of η and *T*. c)Void fraction variations as a function of η and d) void density variations at the end of the simulation along the electrode depth for temperature *T* = 20 °C to *T* = 80 °C. e,f) Li metal surfaces for η = 0.7 V for *T* = 20 °C and *T* = 80 °C respectively.

**Figure** **6** examines how a uniform temperature influences kinetics and transport during lithium stripping. As the temperature increases, the surface diffusion rate and the bulk diffusion rate increase, resulting in faster healing of the interface. In Figure 6a, there is a clear trend of contact area improvement with an increase in temperature from *T* = 20 °C to *T* = 80 °C as a function of η. With an increase in temperature, the overall contribution to the surface diffusion increases, which accelerates the vacancy removal rate from the interface, leading to surface healing. The rough surface morphologies corresponding to *T* = 20 °C and *T* = 80 °C when the overpotential is η 0.7 V are shown in Figure 6e,f. As the temperature elevates, the contact increases from 15% to 55% even higher overpotentials across the interface, leading to smooth solid–solid contact and lower interfacial resistance. In Figure 6b, a contact area map is plotted as a function of η and T showing a clear transition from unstable to stable contact area distribution. Similar trend is also observed below 0 ℃ but the extent of solid‐solid contact is less compared to above zero temperature (Figure S3, Supporting Information). In Figure 6c, the void fraction within Li metal decreases with increasing temperature, as elevated temperatures enhance vacancy transport through the bulk of the electrode. Figure 6d, the void density is more uniformly distributed across the depth of the electrode as a result of high vacancy mobility and an elevated surface diffusion rate. With increasing temperature, vacancies disperse more extensively, forming deeper void structures while mitigating contact loss. In practice, local hot spots can develop because current constrictions occur at partial solid–solid contacts, and interfacial reactions can release or absorb heat. This mechanism is currently out of scope for this model, however, it can be extended to spatially varying temperature by supplying a temperature map and evaluating all event rates with the local temperature using an Arrhenius‐type relation. As vacancy diffusion, surface diffusion, and charge transfer all increase as temperature rises, the qualitative trends reported in Figure 6 are expected to persist under moderate gradients.

## 4 Conclusion

This study develops a mesoscale modeling framework to investigate void formation and interfacial morphology evolution at the Li metal–SE interface. By analyzing the coupled dynamics of vacancy diffusion, surface diffusion kinetics, and interfacial electrochemical reaction, we identify key factors that dictate void growth and solid–solid contact stability. High local reaction rates accelerate vacancy generation and contact degradation, whereas efficient vacancy transport promotes redistribution and surface recovery. We determine the relative influence of surface diffusion modes on the void morphology and interfacial contact distribution, and we conclude that terrace diffusion makes the most contribution to maintaining a smooth surface with high interfacial contact. In contrast, domination of interlayer diffusion increases the roughness of the Li surface and reduces the effective electrochemical contact, raising the interfacial resistance. On the other hand, step diffusion enhances atom‐clustering at the interface, and that's why the absence of step diffusion creates more void fraction inside the Li metal anode. Temperature is shown to be a critical parameter, with an increase from 20 °C to 80 °C improving the active contact area by 40%, due to enhanced Li mobility. We reveal that the competing effect of overpotential with the terrace diffusion creates a clear transition from unstable to stable contact. The roughness factor and the roughness exponent are calculated for three distinct types of Li metal surfaces, and a significant increase in the exponent value is observed, indicating a transition from a smooth surface to a rough surface. In the rough surface scenario, the formation of point contacts amplifies local current density, elevates interfacial polarization, and contributes to rising internal resistance over cycling. Overall, this framework offers mechanistic insight into void dynamics and guides the design of stable Li‐SE interfaces in SSBs.

## Conflict of Interest

The authors declare no conflict of interest.

## Supporting information



Supporting Information

## Data Availability

The data that support the findings of this study are available from the corresponding author upon reasonable request.
