# Supplementary material for: Probing the Impact of Vacancy Diffusion on Void Dynamics at the Lithium Metal–Solid Electrolyte Interface
Source: Adv Sci (Weinh). 2025 Nov 23;13(6):e15827. doi: 10.1002/advs.202515827 (PMC12866773; doi:10.1002/advs.202515827)
Supplement: Supplementary file 1 — Supporting Information [file ADVS-13-e15827-s001.docx]

**Supporting Information**

**Probing the Impact of Vacancy Diffusion on Void Dynamics at the Lithium Metal-Solid Electrolyte Interface**

Sourim Banerjee^1^, Bairav S. Vishnugopi^1^, Partha P. Mukherjee^1,*^

^1^ School of Mechanical Engineering, Purdue University, West Lafayette, IN 47907, United States

**Correspondence:* pmukherjee@purdue.edu

**S1. Coarse-grained mesoscale model**

A coarse-grained mesoscale model is developed to study the dynamic evolution of the electrochemical system. The Kinetic Monte Carlo (KMC) modeling approach incorporates four fundamental mechanisms: metal ion transport in solid-electrolyte, metal atom oxidation at the interface of solid electrolyte (SE) and lithium (Li) metal, bulk diffusion of vacancy from the interface to the bulk of the electrode, and surface diffusion of Li atoms. For each of the processes, a rate constant is first computed at every lattice in the domain. The four rate constants- $k_{T}$ (ion transport rate), $k_{R}$ (electrochemical reaction rate), $k_{D}$ (vacancy transport rate), and $k_{SD}$(surface diffusion rate) are then used to calculate a corresponding total rate constant as follows.

For the transport of metal ions in the electrolyte,

$k_{1}=\sum_{i=1}^{N_{1}} k_{T}^{i}$ (S1)

Here, $N_{1}$ is the number of metal ions in the electrolyte domain, and $k_{1}$ is the total ion transport rate.

For the electrochemical reaction of Li metal atoms,

$k_{2}=\sum_{i=1}^{N_{2}} k_{R}^{i}$ (S2)

Here, $N_{2}$ is the number of metal atoms at the electrode-electrolyte interface and $k_{2}$ is the total electrochemical reaction rate. For the bulk diffusion of a vacancy,

$k_{3}=\sum_{i=1}^{N_{3}} k_{D}^{i}$ (S3)

Here, $N_{3}$ is the number of vacancies in the domain and $k_{3}$ is the total bulk diffusion rate. For the surface diffusion of Li atoms at the Li metal top surface,

$k_{4}=\sum_{i=1}^{N_{4}} k_{SD}^{i}$ (S4)

Here, $N_{4}$ is the number of Li surface atoms going through either terrace diffusion, step diffusion or interlayer diffusion. Based on $k_{1}$, $k_{2}$, $k_{3}$ and $k_{4}$, a total rate constant is then obtained as follows,

$k_{tot}=\sum_{i=1}^{4} k_{i}$ (S5)

A random number $r_{i}$ between 0 to 1 is chosen and is multiplied by $k_{tot}$. Following this, we scan through all the events and select the first event for which the total rate of previously scanned events is larger than ${r_{1}k}_{tot}$. Then, the electrochemical system evolves based on this selected event.

The time step for this event is calculated using another random number $r_{2}$:

$\delta t=-\frac{1}{k_{tot}}ln(r_{2})$ (S6)

A flowchart describing the algorithm used for the dynamic evolution of the electrochemical system is illustrated in Figure S1.


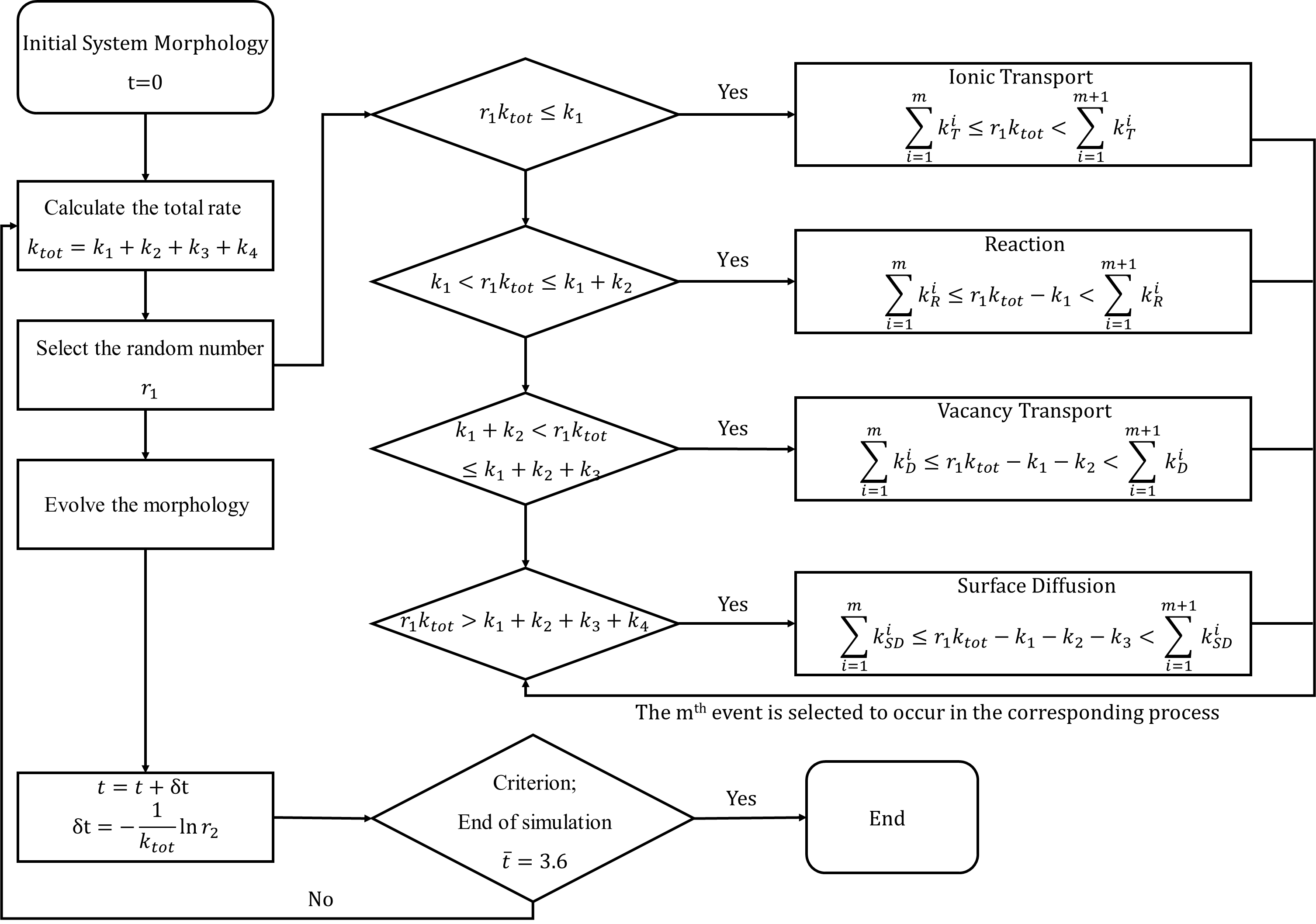


Figure S1. Schematic of the KMC procedure for the dynamics evolution of the electrochemical system

The parameters used in the coarse-grain KMC model have been summarized below in Table S1.

Table S1. Parameters used in the KMC model

|  | Parameters | Values | Units |
| --- | --- | --- | --- |
|  | System dimensions | 50 × 50 × 50 lattice  supercell | - |
| $a_{Li}$ | Lattice size of Li | 3.5 | $Å$ |
| $E_{a,Li^{+}}$ | Ion transport activation barrier^[1]^ | 0.35 | eV |
| $E_{a,vacancy}$ | Vacancy diffusion activation barrier^[2]^ | 0.41 | eV |
| $E_{a,SD, terrace}$ | Terrace surface diffusion activation barrier^[3]^ | 0.3 | eV |
| $E_{a,SD,step}$ | Step surface diffusion activation barrier^[3]^ | 0.15 | eV |
| $E_{a,SD,interlayer}$ | Interlayer surface diffusion activation barrier^[4]^ | 0.5 | eV |
| $F$ | Faraday constant | 96485 | C mol^-1^ |
| $L$ | Domain dimension | 17.5 | nm |
| $\nu$ | Hopping frequency^[5]^ | 2×10^12^ | s^-1^ |
| $k_{b}$ | Boltzmann constant | 8.62×10^-5^ | eV K^-1^ |
| $D_{Li}$ | Bulk diffusivity of Li metal^[6]^ | 2×10^-14^ | m^2^s^-1^ |

**S2. Effect of bulk diffusion on mitigating contact loss**


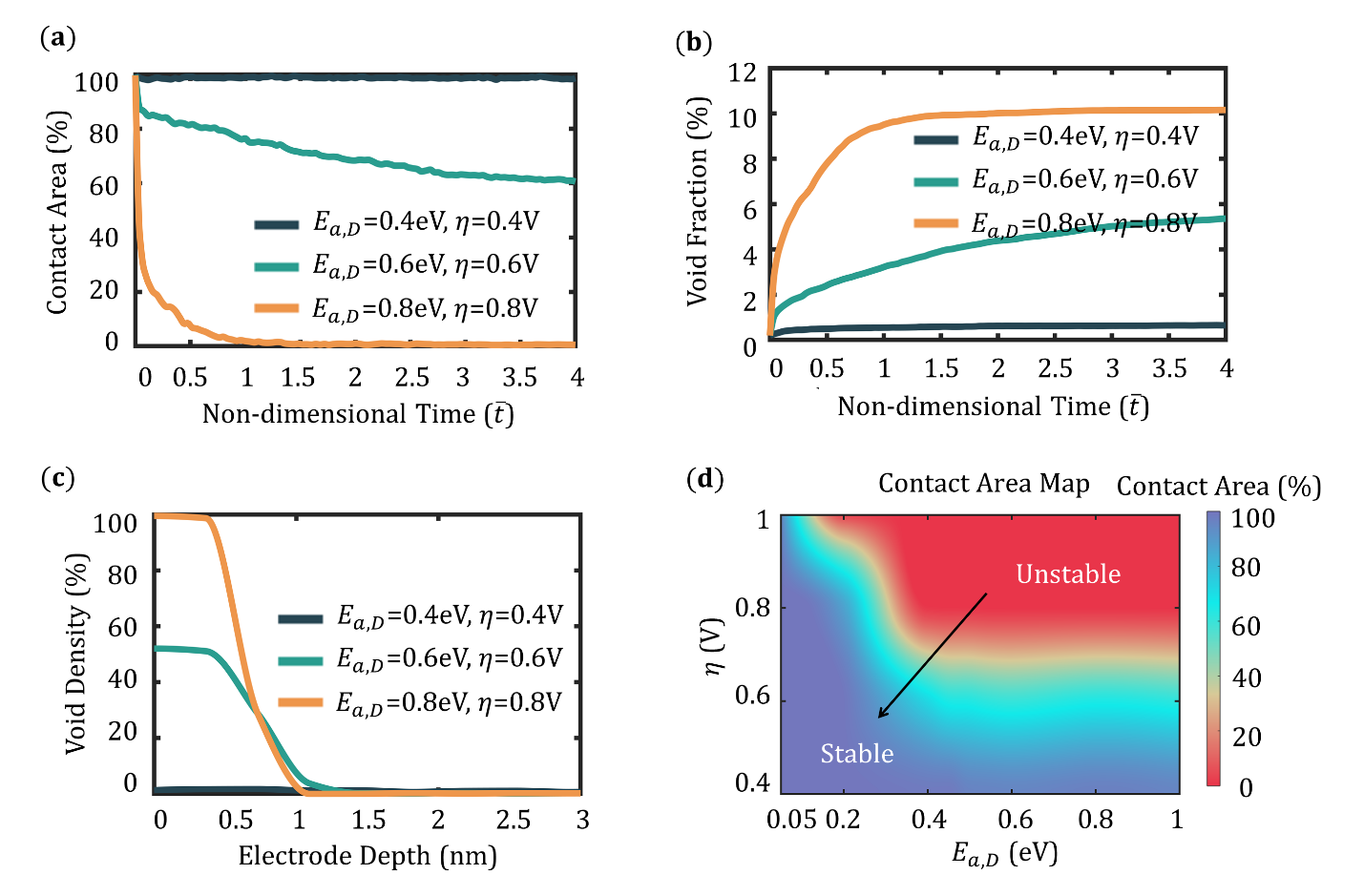


Figure S2. (a) Contact area, (b) void fraction evolution, and (c) final void density distribution along the electrode depth for stable contact ($E_{a,D}$=0.4eV, $\eta$=0.4V), moderate contact ($E_{a,D}$=0.6eV, $\eta$=0.6V), and unstable contact ($E_{a,D}$=0.8eV, $\eta$=0.8V) scenario. (d) Contact area stability map as a function of bulk diffusion activation barrier ($E_{a,D}$) and overpotential ($\eta$).

**S3. Effect of operating temperature in the sub-zero temperature range**


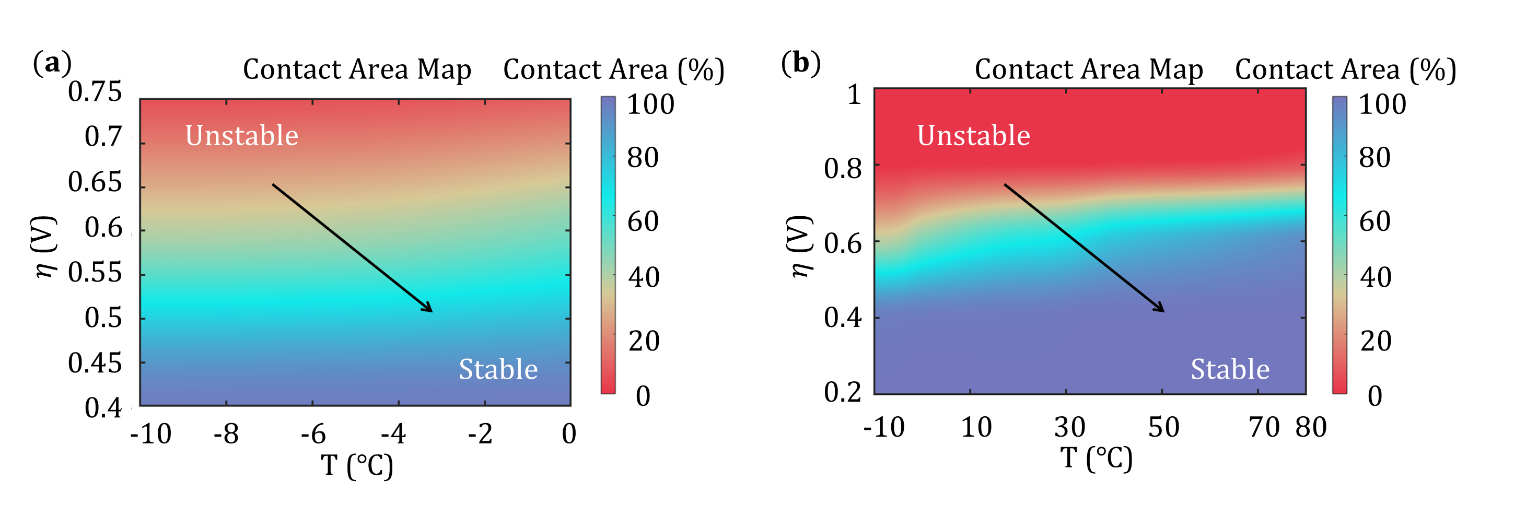


Figure S3. Contact area stability maps between the overpotential ($\eta$) and temperature (T) are shown for (a) sub-zero operating temperature, (b) complete operating temperature range from -10ºC to 80ºC.

**S4. Sensitivity analysis of the surface diffusion activation barriers on contact area, void fraction, and surface roughness factor**


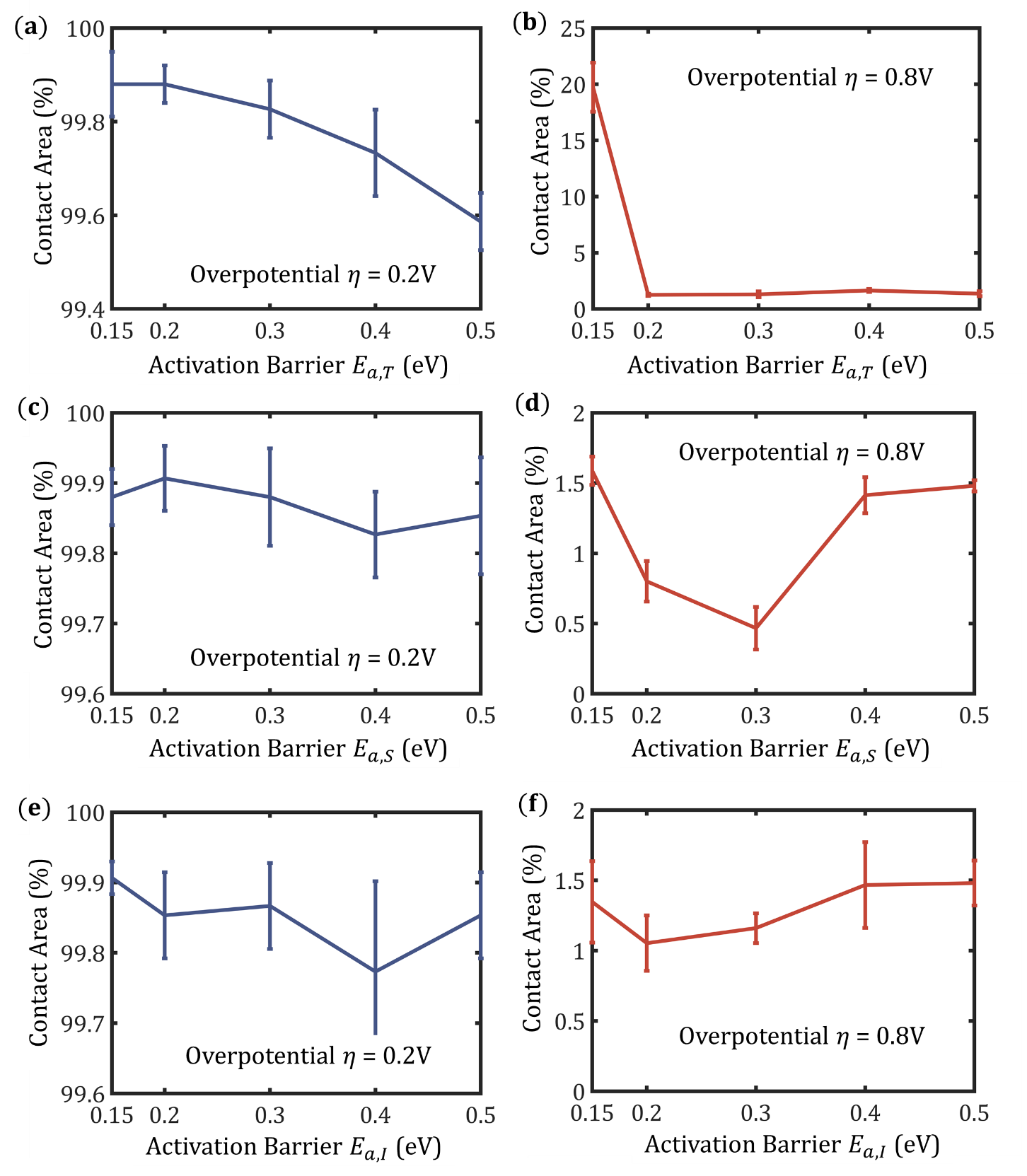


Figure S4. Contact area variation with 95% confidence interval for activation energy variation for terrace diffusion (a)-(b), step diffusion (c)-(d), and interlayer diffusion (e)-(f) for two cases of overpotential values $\eta$=0.2V, and $\eta$=0.8V.


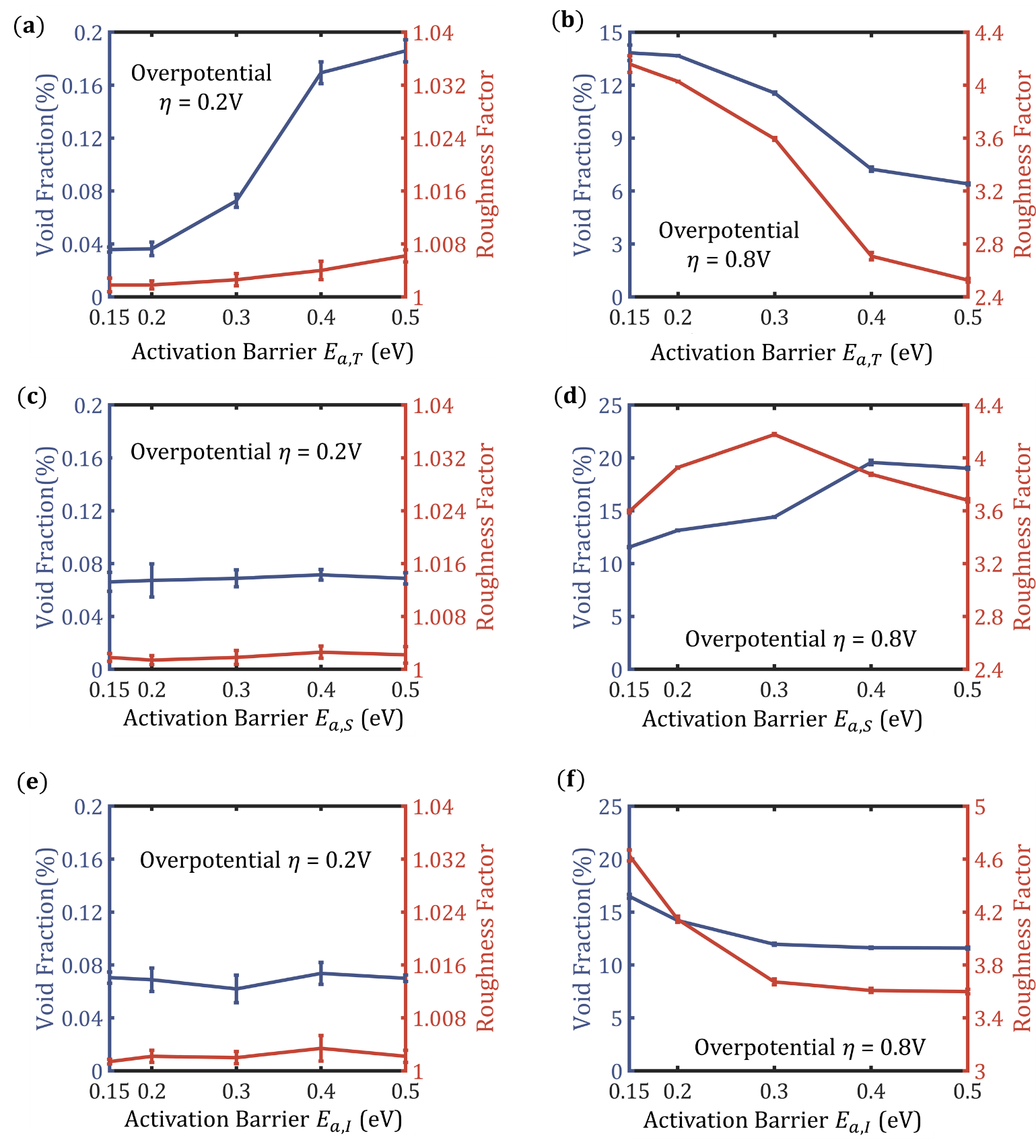


Figure S5. Void fraction and roughness factor variation with 95% confidence interval for activation energy variation for terrace diffusion (a)-(b), step diffusion (c)-(d), and interlayer diffusion (e)-(f) for two cases of overpotential values $\eta$=0.2V, and $\eta$=0.8V.

**References**

[1] B. Andriyevsky, K. Doll, T. Jacob, *Mater. Chem. Phys.* **2017**, *185*, 210–217.

[2] M. Jäckle, A. Groß, *J. Chem. Phys.* **2014**, *141*.

[3] M. Jäckle, K. Helmbrecht, M. Smits, D. Stottmeister, A. Gross, *Energy & Environmental Science* **2018**, *11*, 3400–3407.

[4] M. Jäckle, K. Helmbrecht, M. Smits, D. Stottmeister, A. Groß, *Energy and Environmental Science* **2018**, *11*, 3400–3407.

[5] O. Borodin, G. V. Zhuang, P. N. Ross, K. Xu, *Journal of Physical Chemistry C* **2013**, *117*, 7433–7444.

[6] E. Dologlou, *Glass Phys. Chem* **2010**, *36*, 570–574.
